# The New Generation *h*DHODH Inhibitor MEDS433 Hinders the In Vitro Replication of SARS-CoV-2 and Other Human Coronaviruses

**DOI:** 10.3390/microorganisms9081731

**Published:** 2021-08-14

**Authors:** Arianna Calistri, Anna Luganini, Barbara Mognetti, Elizabeth Elder, Giulia Sibille, Valeria Conciatori, Claudia Del Vecchio, Stefano Sainas, Donatella Boschi, Nuria Montserrat, Ali Mirazimi, Marco Lucio Lolli, Giorgio Gribaudo, Cristina Parolin

**Affiliations:** 1 Department of Molecular Medicine, University of Padua, 35121 Padua, Italy; arianna.calistri@unipd.it (A.C.); valeria.conciatori@gmail.com (V.C.); claudia.delvecchio@unipd.it (C.D.V.); cristina.parolin@unipd.it (C.P.); 2 Department of Life Sciences and Systems Biology, University of Turin, 10123 Turin, Italy; anna.luganini@unito.it (A.L.); barbara.mognetti@unito.it (B.M.); giulia.sibille@unito.it (G.S.); 3 Public Health Agency of Sweden, 17182 Solna, Sweden; elizabeth.elder@folkhalsomyndigheten.se (E.E.); ali.mirazimi@folkhalsomyndigheten.se (A.M.); 4 Department of Sciences and Drug Technology, University of Turin, 10125 Turin, Italy; stefano.sainas@unito.it (S.S.); donatella.boschi@unito.it (D.B.); marco.lolli@unito.it (M.L.L.); 5 Pluripotency for Organ Regeneration, Institute for Bioengineering of Catalonia (IBEC), The Barcelona Institute of Technology (BIST), 08028 Barcelona, Spain; nmontserrat@ibecbarcelona.eu; 6 Catalan Institution for Research and Advanced Studies (ICREA), 08010 Barcelona, Spain; 7 Centro de Investigación Biomédica en Red en Bioingeniería, Biomateriales y Nanomedicina, 28029 Madrid, Spain; 8 Karolinska Institute and Karolinska University Hospital, Department of Laboratory Medicine, Unit of Clinical Microbiology, 17177 Stockholm, Sweden; 9 National Veterinary Institute, 75189 Uppsala, Sweden

**Keywords:** coronavirus, SARS-CoV-2, pyrimidine biosynthesis, *h*DHODH inhibitor, antiviral activity, combination treatment, organoids, broad-spectrum antiviral

## Abstract

Although coronaviruses (CoVs) have long been predicted to cause zoonotic diseases and pandemics with high probability, the lack of effective anti-pan-CoVs drugs rapidly usable against the emerging SARS-CoV-2 actually prevented a promptly therapeutic intervention for COVID-19. Development of host-targeting antivirals could be an alternative strategy for the control of emerging CoVs infections, as they could be quickly repositioned from one pandemic event to another. To contribute to these pandemic preparedness efforts, here we report on the broad-spectrum CoVs antiviral activity of MEDS433, a new inhibitor of the human dihydroorotate dehydrogenase (*h*DHODH), a key cellular enzyme of the de novo pyrimidine biosynthesis pathway. MEDS433 inhibited the in vitro replication of hCoV-OC43 and hCoV-229E, as well as of SARS-CoV-2, at low nanomolar range. Notably, the anti-SARS-CoV-2 activity of MEDS433 against SARS-CoV-2 was also observed in kidney organoids generated from human embryonic stem cells. Then, the antiviral activity of MEDS433 was reversed by the addition of exogenous uridine or the product of *h*DHODH, the orotate, thus confirming *h*DHODH as the specific target of MEDS433 in hCoVs-infected cells. Taken together, these findings suggest MEDS433 as a potential candidate to develop novel drugs for COVID-19, as well as broad-spectrum antiviral agents exploitable for future CoVs threats.

## 1. Introduction

We have been living with COVID-19 for more than a year and a half. One of the most dramatic things this pandemic has taught us is the total unpreparedness to tackle a novel coronavirus disease by a curative approach. Despite the fact that coronaviruses (CoVs) have long been predicted to cause zoonotic diseases with high probability, as SARS and MERS outbreaks demonstrated, the lack of effective pan-coronavirus (pan-CoVs) antivirals significantly contributed to the vulnerability of our public health systems to the SARS-CoV-2 pandemic, thus making the therapeutic management of COVID-19 mostly supportive, with the only aim to reduce mortality [1,2,3].

It is reassuring that prevention of COVID-19 is now achievable thanks to the deployment of effective next generation SARS-CoV-2-specific vaccines [4]. However, even in the best scenario possible, we must not fail to consider that: (1) the evolution of SARS-CoV-2 is leading to virus variants that might escape the antibody-mediated immunity and cause reinfections; (2) the durability of protection afforded by the vaccines is not yet known and it may be limited; (3) the large scale-immunization to achieve a global vaccine coverage may not be complete due to unfolding productive and logistical challenges and reluctance to vaccinate; and (4) the value of the current SARS-CoV-2 specific vaccines for effective prevention of new CoVs that might likely may emerge in future from animal reservoir hosts is unknown [5].

For all these reasons, effective broad-spectrum anti-CoVs antivirals can be highly valuable to fill the gap in the control of emerging CoVs diseases given their rapid repositioning from one pandemic event to another. This will allow us to: (1) protect individuals with susceptibility to severe disease from new CoVs and thus save lives; (2) contribute to Public Health measures by slowing down the spread of the new disease; and (3) buy time while waiting for CoV-specific vaccine development [6,7].

To be candidate broad-spectrum anti-CoVs agents, small molecules must interfere with host or viral targets that are: (1) highly conserved among known CoVs; (2) essential to replication or pathogenesis of known hCoVs; and (3) likely to be conserved and essential even in future emerging CoVs [7]. To develop such a molecule, two main strategies can be followed. The first aims to discover broad spectrum Direct Acting Antivirals (DAAs) that target the most conserved proteins among CoVs, such as the RNA-dependent RNA polymerase, the 3C-like protease or main protease (M^pro^), and the papain-like Protease (PL^pro^) [8,9]. However, targeting such viral proteins by DAAs is prone to the development of viral resistance, as is well established for other RNA viruses. Therefore, the second drug development strategy works toward the design of broad-spectrum anti-CoVs agents able to interfere with those cellular enzymes and factors utilized by all CoVs during their life cycle. Clearly, these Host-Targeting Antivirals (HTAs) can overcome the potential emergence of drug resistance associated with DAAs, but have the disadvantages of potential off-target effects [8,9].

Pyrimidine availability in infected cells is crucial for CoVs replication and thus compounds targeting the cellular de novo pyrimidine biosynthetic pathway have the potential to be developed as effective broad-spectrum anti-CoV HTA agents. In this biochemical pathway, the dihydroorotate dehydrogenase (DHODH) catalyzes the rate-limiting step of dehydrogenation of dihydroorotate (DHO) to orotate (ORO), ultimately providing uridine monophosphate (UMP), the precursor for all pyrimidine nucleotides for RNA and DNA [10,11,12,13]. Therefore, given its critical role, DHODH could be a target of choice for the development of HTAs against SARS-CoV-2 and CoVs and, to validate this antiviral strategy, potent and safe human DHODH (*h*DHODH) inhibitors are urgently required [14]. In this regard, starting from the scaffold of brequinar, one of the most potent *h*DHODH inhibitors so far discovered [15], we have contributed to this need by developing a novel class of potent *h*DHODH inhibitors characterized by an unusual carboxylic bioisostere 2-hydroxypyrazolo [1,5-a] pyridine moiety [16,17].

The aim of this study was thus to investigate the potential of this new class of *h*DHODH inhibitors as broad-spectrum CoVs antivirals. We report that one of these molecules, termed MEDS433, potently inhibits the in vitro replication of SARS-CoV-2 and other hCoVs through the selective block of *h*DHODH enzymatic activity. These results identify MEDS433 as a promising candidate to develop new broad-spectrum anti-CoVs agents.

## 2. Materials and Methods

### 2.1. Compounds

**Figure 1:**
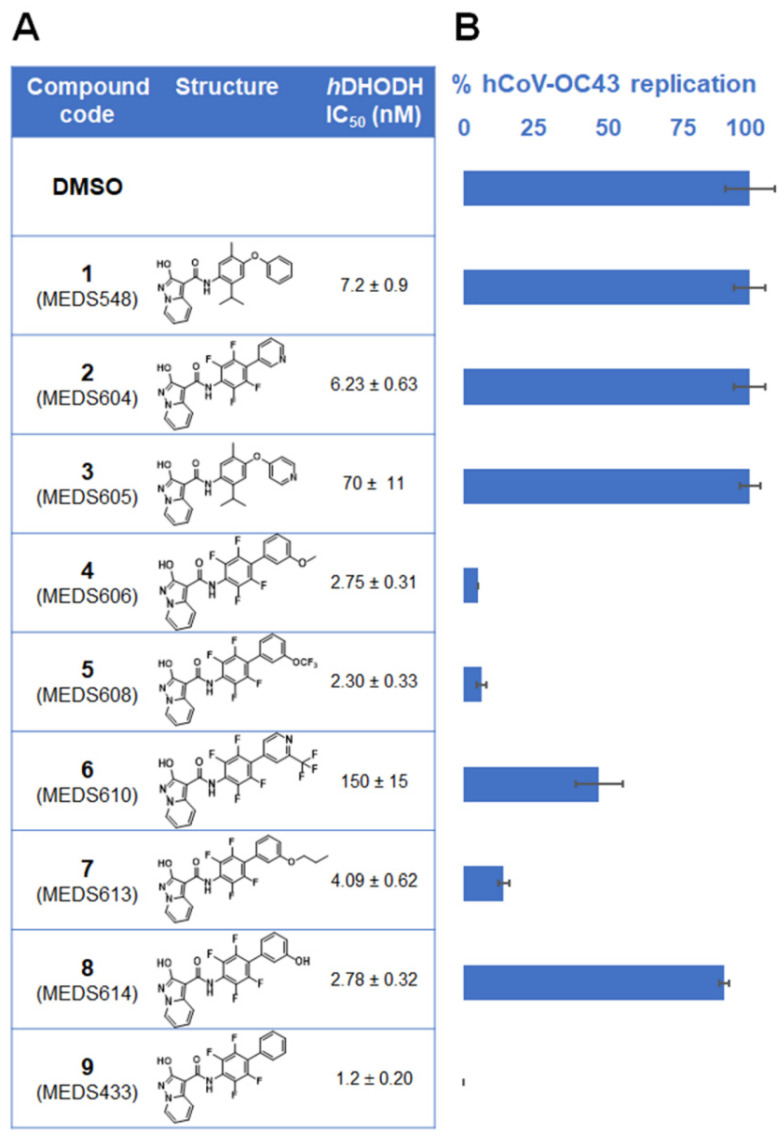
Selection of *h*DHODH inhibitors with anti-hCoV activity. HCT-8 cells were pretreated and treated vehicle (DMSO) or with 0.1 μM of the different *h*DHODH inhibitors showed in (**A**) 1 h prior to, during infection with hCoV-OC43 (100 PFU/well), and throughout the experiment. At 72 h p.i., viral foci were immunostained and the mean foci number in treated culture compared to that of DMSO-treated and hCoV-OC43-infected control HCT-8 cell monolayers. The replication rates shown in (**B**) represent means ± SD (error bars) of three independent experiments performed in triplicate.

The *h*DHODH inhibitors (Figure 1A) were synthesized as described previously [16,17]. Brequinar, uridine, orotic acid (ORO), dihydroorotic acid (DHO), and dipyridamole (DPY) were purchased from Sigma-Aldrich. Remdesivir (RDV) (GS-5734) was obtained by MedChemExpress.

### 2.2. Cells and Viruses

Human lung fibroblasts MRC5 (ATCC CCL-171), the human colorectal carcinoma HCT-8 (ATCC CCL-244), the human lung adenocarcinoma Calu-3 (ATCC HTB-55), and the African green monkey kidney Vero E6 (ATCC CRL-1586) cell lines were purchased from the American Type Culture Collection (ATCC), and maintained in Dulbecco’s Modified Eagle Medium (DMEM; Euroclone) supplemented with 10% fetal bovine serum (FBS, Euroclone), 2 mM glutamine, 1 mM sodium pyruvate, 100 U/mL penicillin, and 100 μg/mL streptomycin sulfate (P/S, both from Euroclone).

hCoV-229E (ATCC VR-740) and hCoV-OC43 (ATCC VR-1558) were purchased from ATCC, and propagated and titrated in MRC5 and HCT-8 cells, respectively. SARS-CoV-2 (2019-nCoV/Italy INMI1) was obtained from EVAg, and propagated and titrated in Vero E6 cells. SARS-CoV-2/01/human/2020/SWE was isolated on Vero E6 cells from a nasopharyngeal sample, cultivated and titrated as previously described [18]. All work with SARS-CoV-2 was performed in Biosafety laboratory level 3 (BSL3) facilities either at the University of Padua, Italy, or at the Public Health Agency of Sweden, Sweden.

### 2.3. Cytotoxicity Assays

Cells were seeded in 96-well plates and, after 24 h, exposed to increasing concentrations of compounds or vehicle (DMSO) as control. After 72 h of incubation, the number of viable cells was determined using either the CellTiter-Glo Luminescent assay (Promega, Madison, WI, USA) according to the specifications of the manufacturer, or the MTT method as previously described [19,20].

### 2.4. Antiviral Assays

To select the mini-library of *h*DHODH inhibitors, focus forming reduction assays (FFRAs) [21] were performed on HCT-8 cell monolayers treated with the vehicle (DMSO) or with 0.1 μM of the different compounds 1 h prior to and during infection with the hCoV-OC43 (100 PFU/well). At 72 h post-infection (p.i.), cell monolayers were fixed, and subjected to indirect immunoperoxidase staining with a mAb against the hCoV-OC43 N protein (clone.542-D7; Millipore, Burlington, MA, USA) (diluted 1:100). Viral foci were microscopically counted, and the mean counts for each drug concentration were expressed as a percentage of the mean plaque counts of control virus (DMSO). To determine the anti-hCoV-229E activity of MEDS433 or brequinar, MRC5 cell monolayers were treated with different concentrations of the compounds 1 h prior to and during infection with hCoV-229E (100 PFU/well). After 72 h p.i., cell viability was measured using CellTiter-Glo assay as a surrogate measurement of the viral cytopathic effect (CPE), as previously described [22]. To measure the anti-SARS-CoV-2 activity of MEDS433 or brequinar, virus yield reduction assay (VRA) was performed with Vero E6 or Calu-3 cells. Briefly, cell monolayers were treated with the vehicle or increasing concentrations of compounds 1 h before and during infection with SARS-CoV-2 (50 or 100 PFU/well). At 48 h p.i., SARS-CoV-2 in cell supernatants was titrated by plaque assay on Vero E6 cells. Compound concentrations producing 50 and 90% reductions in plaque formation (EC_50_ and EC_90_) were determined as compared to control treatment (DMSO). To evaluate the effect of uridine, DHO or ORO addition, VRAs were performed with Vero E6 or Calu-3 cells infected with SARS-CoV-2 (50 or 100 PFU/well), and treated with increasing concentrations of uridine, ORO or DHO in presence of 0.3 μM of MEDS433 for Vero E6 cells or 0.5 μM for Calu-3 cells. After 48 h p.i., cell supernatants were harvested and titrated for SARS-CoV-2 infectivity on Vero E6 cells.

To assess the effect of blocking both the de novo biosynthesis and the salvage pathway of pyrimidines, Calu-3 cells were infected with SARS-CoV-2 (100 PFU/well) and treated throughout VRAs with different concentration of MEDS433 in combination with increasing concentrations of DPY in medium supplemented with uridine (20 μM). At 48 h p.i., supernatants were harvested and titrated for SARS-CoV-2 infectivity on Vero E6 cells.

To measure the effect of MEDS433 on SARS-CoV-2 intracellular RNA load, Calu-3 cells were seeded in 24 well-plates and 24 h later were treated with the vehicle or 0.5 µM of compound 1 h before, and during infection with SARS-CoV-2 (100 PFU/well). At 10, 24, and 48 h p.i., total RNA was extracted from cell pellets (PureLink™ RNA Mini Kit-Thermo Fisher Scientific, Waltham, MA, USA) and quantified by Nanodrop 1000 spectrometer (Thermo Scientific). Next, 500 ng RNA were retrotranscribed by means of the High capacity cDNA reverse transcription kit (Applied Biosystem, Waltham, MA, USA) and 50 ng of cDNA were amplified by qPCR by using the following SARS-CoV-2 N gene specific primers/probes [23]:

Forward: CACATTGGCACCCGCAATC;

Reverse: GAGGAACGAGAAGAGGCTTG;

Probe: FAM-ACTTCCTCAAGGAACAACATTGCCA-BBQ.

Data were normalized using GAPDH (ThermoFisher) and analyzed with the 2^−ΔΔC^_t_ method [24] by adopting the mean of the ΔC_t_ values obtained for the vehicle treated samples as reference. Finally, data were expressed as the ratio between values calculated for MEDS433 treated samples, and the mean of the ones calculated for vehicle treated samples ± standard deviation.

### 2.5. Kidney Organoid Generation and SARS-CoV-2 Infection

Kidney organoids were differentiated from human embryonic stem cells as previously described [18,25], and infected with 100 PFU/organoid of SARS-CoV-2/01/human/2020/SWE [18] at day 20–25 of differentiation in advanced RPMI medium (ThermoFisher). Organoids were infected in the presence of DMSO or 0.5 µM MEDS433 in ultra-low attachment plates for 48 h. Organoids were then pooled into groups of four, washed three times with PBS and lysed in Trizol. RNA was extracted using Direct Zol RNA mini kit (Zymo research), and analyzed for the content of SARS-CoV-2 RNA by RT-qPCR using the following primers/probes as previously described [18]:

SARS-CoV-2 E gene:

Forward: ACAGGTACGTTAATAGTTAATAGCGT;

Reverse: ATATTGCAGCAGTACGCACACA;

Probe: FAM-ACACTAGCCATCCTTACTGCGCTTCG-MGB.

Human RNase P:

Forward: AGATTTGGACCTGCGAGCG;

Reverse: GAGCGGCTGTCTCCACAAGT;

Probe: VIC-TTCTGACCTGAAGGCTCTGCGCG-MGB.

Human RNase P was used as an endogenous gene control to normalize the levels of intracellular SARS-CoV-2 RNA.

### 2.6. Immunofluorescence

Vero E6 cells seeded on coverslip were treated with vehicle or with 0.5 μM MEDS433 1 h prior to infection with SARS-CoV-2 at an MOI of 0.1. At 24 h p.i., cells were fixed, permeabilized, and stained with an anti-SARS-CoV-2 N nucleocapsid protein mAb (Sino Biologicals, Beijing, China) followed by Alexa 488-conjugated rabbit anti-mouse antibody (Life Technologies, Carlsbad, CA, USA). Nuclei were stained with DRAQ5 (InVitrogen, Waltham, MA, USA).

### 2.7. Statistical Analysis

All statistical analyses were performed using GraphPad Prism version 7.0. Data of antiviral activity are presented as the means ± SDs of at least three experiments performed in triplicate. Differences were considered to be statistically significant for *p* < 0.05.

## 3. Results

### 3.1. Identification of a New hDHODH Inhibitor Active against a Human Coronavirus

To investigate the feasibility of targeting *h*DHODH activity to develop broad-spectrum HTA, nine newly designed *h*DHODH inhibitors (Figure 1A) [16,17] were selected for antiviral activity against the prototypical human β-CoV, hCoV-OC43, by using FFRAs in which test compounds were present before, during and after infection (full-treatment). As shown in Figure 1B, when tested at 0.1 μM, five *h*DHODH inhibitors (4, 5, 6, 7, and 9) were able to decrease hCoV-OC43 replication more than 50%, while the remaining four compounds (1, 2, 3 and 8) were ineffective. Notably, compound 9, MEDS433, was the most effective among the tested *h*DHODH inhibitors, since it abrogated hCoV-OC43 replication completely (Figure 1B). MEDS433 was therefore selected for further investigations.

### 3.2. Inhibition of α- and β-Coronavirus Replication by the hDHODH Inhibitor MEDS433

**Figure 2:**
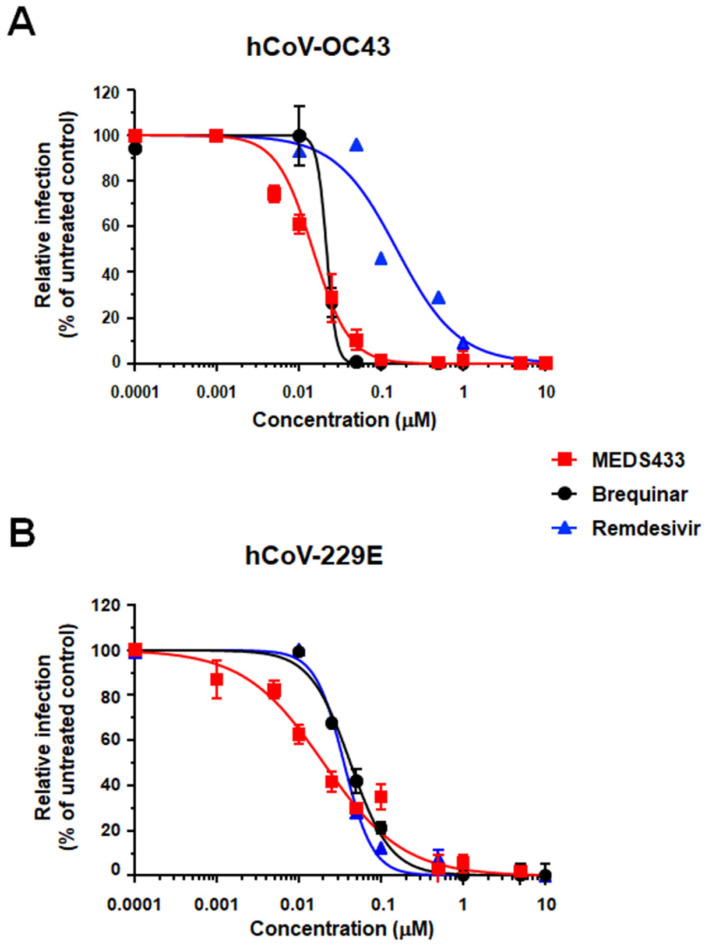
Antiviral activity of MEDS433 against hCoV-OC43 and hCoV-229E. HCT-8 (**A**) or MRC5 (**B**) cell monolayers were infected with the hCoV-OC43 or hCoV-229E (100 PFU/well), and, where indicated, the cells were treated with increasing concentrations of compounds 1 h before, and during virus adsorption. Compounds remained in the culture medium throughout the experiment. hCoV-OC43 replication was quantified at 72 h p.i. by FFRA, while for hCoV-229E, MRC5 cell viability was measured using the CellTiter-Glo luminescent at 72 h p.i. as a surrogate of viral CPE. The compounds concentrations producing 50% and 90% reductions of viral replication (EC_50_ and EC_90_, respectively) were determined by GraphPad Prism. The data shown represent means ± SD (error bars) of three independent experiments performed in triplicate.

A significant concentration-dependent inhibition of hCoV-OC43 replication was then confirmed in HCT-8 cells treated with MEDS433 (Figure 2A). MEDS433 was also very effective against another hCoV, the prototypic α-hCoV-229E, whose replication in MRC5 fibroblasts was severely impaired (Figure 2B). As reported in Table 1, the measured EC_50_ and EC_90_ values for both hCoVs were in the low nanomolar range. The comparison with the reference drug RDV, used as a positive control for anti-hCoV antiviral activity [19], highlighted an anti-hCoV-229E potency of MEDS433 comparable to that of RDV (EC_50_ 0.0348 ± 0.005 μM), while the *h*DHODH inhibitor was much more effective than RDV (EC_50_ 0.147 ± 0.034 μM) against hCoV-OC43. To the contrary, MEDS433 was more effective than brequinar against hCOV-229E, as the EC_50_ of the latter was 0.0427 ± 0.003 μM, whereas against hCoV-OC43 the EC_50_ of brequinar (0.022 ± 0.003 μM) was commensurate with that of MEDS433. Finally, the anti-hCoVs activity of MEDS433 was not due to cytotoxicity of target cells themselves, since its cytotoxic concentration (CC_50_) as determined in uninfected cells was 78.48 ± 4.6 μM for HCT-8 cells, and 104.80 ± 19.75 μM for MRC5 fibroblasts, with a favorable Selective Index (SI) greater than 6300 and 4600 for hCoV-OC43 and hCoV-299E, respectively (Table 1).

### 3.3. MEDS433 Exerts an Antiviral Activity against SARS-CoV-2

**Figure 3:**
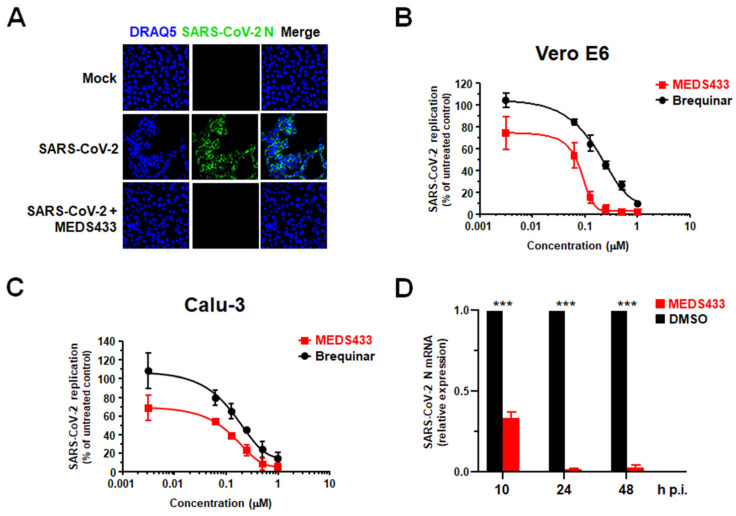
Antiviral activity of MEDS433 against SARS-CoV-2 replication. (**A**) Immunofluorescence analysis of SARS-CoV-2-infected cells. Vero E6 cells were treated with vehicle (DMSO) or with 0.5 μM MEDS433 1 h prior to infection with SARS-CoV-2 at an MOI of 0.1. At 24 h p.i., cells were fixed, permeabilized, and immunostained with an anti-SARS-CoV-2 nucleocapsid protein (N) mAb, followed by Alexa 488-conjugated secondary antibody. Nuclei were stained with DRAQ5. Confocal laser microscopy images acquired in the green (SARS-CoV-2 N) and the blue (DRAQ5) channels are shown, as well as overlaid images (merge). Magnification, ×60. (**B**) Dose dependent inhibition of SARS-CoV-2 replication by MEDS433 in Vero E6 cells. Vero E6 cell monolayers were infected with SARS-CoV-2 (50 PFU/well), and, where indicated, the cells were treated with vehicle (DMSO) or increasing concentrations of MEDS433 or brequinar 1 h before, during virus adsorption, and throughout the experiment. At 48 h p.i., infectious SARS-CoV-2 in cell supernatants was titrated by plaque assay on Vero E6 cells. (**C**) Anti-SARS-CoV-2 activity of MEDS433 in Calu-3 cells. Calu-3 cells were infected with SARS-CoV-2 (100 PFU/well), and, where indicated, the cells were treated with vehicle (DMSO) or increasing concentrations of MEDS433 or brequinar 1 h before, during virus adsorption, and throughout the experiment. At 48 h p.i., infectious SARS-CoV-2 in cell supernatants was titrated by plaque assay on Vero E6 cells. MEDS433 and brequinar concentrations producing 50 and 90% reductions in SARS-CoV-2 yield (EC_50_ and EC_90_, respectively) were determined as compared to control treatment (DMSO). The data shown represent means ± SD (error bars) of three independent experiments performed in triplicate. (**D**) Effect of MEDS433 on SARS-CoV-2 intracellular RNA. Calu-3 cells were treated with vehicle (DMSO) or 0.5 µM of MEDS433 1h before and during infection with SARS-CoV-2 (100 PFU/well). At 10, 24, and 48 h p.i., total RNA was extracted and subjected to RT-qPCR by using primers and probe specific for the SARS-CoV-2 N gene. Data were normalized using GAPDH and analyzed with the 2^−ΔΔC^_t_ method by adopting the mean of the ΔC_t_ values obtained for the vehicle treated samples as reference. The reported values represent that ratio between data calculated for MEDS433 treated samples and the mean of the ones calculated for vehicle treated samples ± standard deviation. Statistical significance was calculated by a one-way ANOVA followed by Dunnett’s multiple comparison test. *** (*p* < 0.0001) compared to the calibrator sample (DMSO).

To investigate the effect of MEDS433 on SARS-CoV-2 replication, Vero E6 cells were infected with a clinical isolate of SARS-CoV-2, and then treated with 0.5 μM MEDS433. At 24 h p.i., cells were fixed, permeabilized and immunostained for the nucleocapsid N protein expression. As shown in Figure 3A, confocal microscopy revealed that while about 85% of infected control cells expressed the N protein, MEDS433 treatment completely abolished its accumulation, thus indicating that N protein expression could be prevented by targeting the de novo pyrimidine biosynthesis.

VRAs were then performed in SARS-CoV-2-infected Vero E6 cells treated with increasing concentrations of MEDS433 for 48 h and a clear concentration-dependent inhibition of SARS-CoV-2 replication was measured (Figure 3B). Of note, the anti-SARS-CoV-2 activity of MEDS433 was also observed in the more relevant cell model of the human lung adenocarcinoma Calu-3 cells (Figure 3C), in which of 0.076 ± 0.005 μM and 0.513 ± 0.016 μM were measured. Again, the measured EC_50_ and EC_90_ values of MEDS433 in both Vero E6 and Calu-3 cell models were in the low nanomolar range (Table 1). Furthermore, MEDS433 was more effective than brequinar against SARS-CoV-2, since the EC_50_ values of brequinar were of 0.200 ± 0.01 μM in Vero E6 cells, and of 0.214 ± 0.002 μM in Calu-3 cells, respectively (Figure 3B,C). Then, as shown in Table 1, the CC_50_ values of MEDS433 measured in uninfected cells, were >500 μM in Vero E6 cells (SI > 7900) and >125 μM in Calu-3 cells (SI > 1600), thus indicating that its anti-SARS-CoV-2 activity was not due to a reduced cell viability.

Finally, to evaluate a direct effect of *h*DHODH inhibition on the RNA synthesis step of SARS-CoV-2 replicative cycle, the intracellular accumulation of viral N transcript was measured in infected Calu-3 cells treated with 0.5 µM of MEDS433 for 10, 24 or 48 h p.i. As depicted in Figure 3D, RT-qPCR quantification showed a significant reduction in N mRNA levels already at 10 h p.i., and this inhibition became more severe at later times of infection, when MEDS433 almost completely suppressed N mRNA synthesis, thus confirming the importance of the *h*DHODH activity for efficient SARS-CoV-2 replication.

Taken together, the results of the different antiviral assays against SARS-CoV-2, as well as those against hCoV-229E and hCoV-OC43 (Figure 2), highlight a broad-spectrum anti-CoVs activity of MEDS433.

### 3.4. MEDS433 Affects SARS-CoV-2 Replication in Kidney Organoids

Organoids set a pattern of the physiological conditions of human organs, and therefore they can be suitable infection models for the development of anti-SARS-CoV-2 drugs [26]. Relevant to a suitable organoids model, it is known that, in addition to the lung damage caused by pneumonia, SARS-CoV-2 infection affects several other organs like the kidney. Severe COVID-19 disease is in fact frequently associated with kidney injury produced by both indirect mechanisms and direct damage due to virus replication [27,28]. Actually, SARS-CoV-2 has been found in autopsy specimens and urine samples [29,30], and its cellular receptor, ACE2, is highly expressed in renal tubules [31].

**Figure 4:**
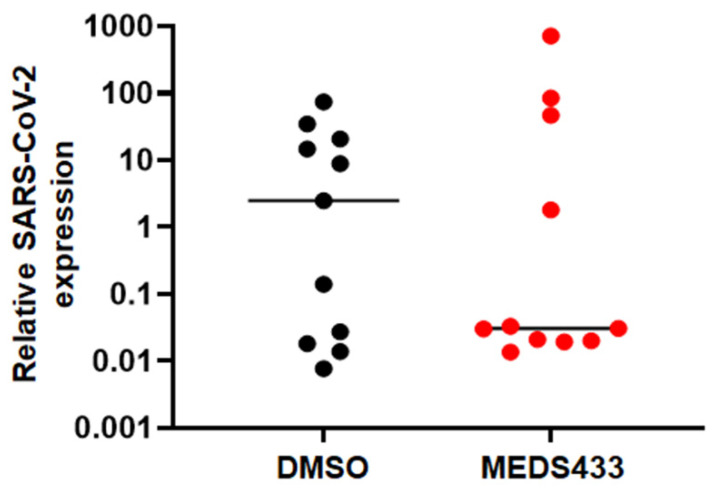
Effects of MEDS433 on SARS-CoV-2 replication in kidney organoids. Kidney organoids were infected with 100 PFU of SARS-CoV-2/01/human/2020/SWE per organoid and simultaneously treated with 0.5 µM MEDS433 or vehicle (DMSO) as a control. After 48 h, kidney organoids were washed, and harvested for analysis of SARS-CoV-2 E RNA levels by RT-qPCR. The results from three independent batches of kidney organoids are shown with line at mean relative to mock-infected controls; batches 1 and 2 contained 3 groups of 4 pooled organoids, batch 3 contained 5 groups of 4 pooled organoids per treatment condition.

Based on this rationale, to analyze the effect of MEDS433 in a more physiologically and relevant in vitro system, we used kidney organoids as a model of SARS-CoV-2 infection [18]. The organoids used in our model are heterogeneous, and therefore multiple batches of kidney organoids differentiated from human stem cells were employed to perform the antiviral assays. As shown in Figure 4, the average results obtained from infections of three separate batches of organoids indicated that the inhibitory effect of MEDS433 on SARS-CoV-2 replication could be reproduced in kidney organoids, thus suggesting an anti-SARS-CoV-2 activity of MEDS433 even in this complex experimental model.

### 3.5. The Antiviral Activity of MEDS433 Is Reversed by Uridine and Orotic Acid

**Figure 5:**
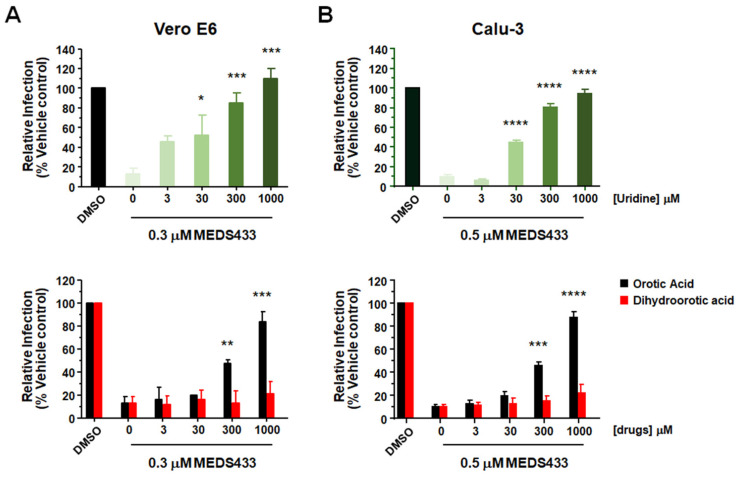
Uridine or orotic acid supplementation counteracts the anti-SARS-CoV-2 activity of MEDS433. Vero E6 (**A**) or Calu-3 (**B**) cells were treated with vehicle (DMSO), or 0.3 μM of MEDS433 for Vero E6 cells, or 0.5 μM of MEDS433 for Calu-3 cells in the absence or presence of increasing concentrations of uridine (upper panel), orotic acid or dihydroorotic acid (lower panel) before and during infection with SARS-CoV-2 (50 PFU/well for Vero E6 cells and 100 PFU for Calu-3 cells). At 48 h p.i. cell supernatants were harvested and titrated on Vero E6 cells. Plaque counts for each drug concentration were expressed as a percent of the mean count of the control cultures treated with DMSO. The data shown represent means ± SD of three independent experiments performed in triplicate. Statistical significance was calculated by a one-way ANOVA followed by Dunnett’s multiple comparison test, **** (*p)* < 0.00001), *** (*p* < 0.0001), ** (*p* < 0.001) and * (*p* < 0.05) compared to the calibrator sample (MEDS433 alone).

To verify the hypothesis of an interference with the pyrimidine biosynthesis pathway as the mechanism of the anti-hCoVs activity of MEDS433, we investigated in Vero E6 and Calu-3 cells whether the anti-SARS-CoV-2 activity of MEDS433 could be overcome by supplementing cell medium with increasing concentrations of exogenous uridine, thus bypassing the requirement of de novo pyrimidine biosynthesis. As shown in Figure 5, the anti-SARS-CoV-2 activity of 0.3 μM MEDS433 in Vero-E6 (upper panel A) and of 0.5 μM in Calu-3 (upper panel B) cells was significantly reversed by a 100-fold excess of uridine relative to MEDS433 concentration, and completely overturned by greater uridine concentrations, thus confirming that the de novo pyrimidine pathway was inhibited by MEDS433 in SARS-CoV-2-infected cells. Then, to prove that *h*DHODH inhibition was responsible of MEDS433 antiviral effect, increasing concentrations of the *h*DHODH substrate dihydroorotic acid or its product, orotic acid were added to cell medium. In Vero E6 or Calu-3 cells treated with MEDS433 and infected with SARS-CoV-2, the addition of orotic acid reversed in a dose dependent manner the antiviral effect of MEDS433 (Figure 5 lower panels), with complete reversion achieved at the highest concentration (1000× the MEDS433 concentration). In contrast, dihydroorotic acid, even at 1 mM (3333 times more than MEDS433), did not affect MEDS433 antiviral activity (Figure 5, lower panels), thus indicating that MEDS433 inhibited a step in the de novo pyrimidine biosynthesis pathway downstream from dihydroorotic acid.

Altogether, these results confirmed that MEDS433 specifically targets *h*DHODH activity in SARS-CoV-2-infected cells, and that this inhibition is responsible of its overall antiviral activity.

### 3.6. A Combination of MEDS433 with an Inhibitor of the Pyrimidine Salvage Pathway Is Effective against SARS-CoV-2 Replication Even in the Presence of Uridine

**Figure 6:**
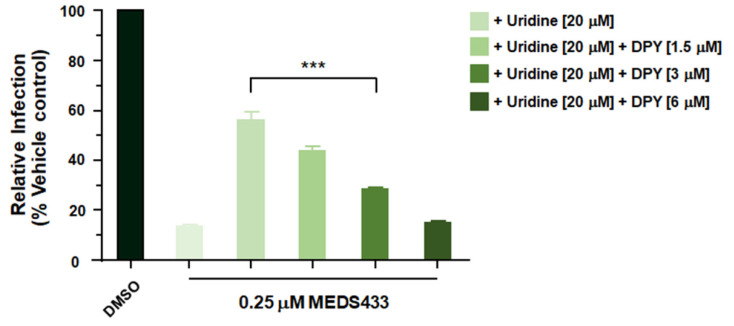
A combination of MEDS433 and dipyridamole hampers SARS-CoV-2 replication even in the presence of uridine. Calu-3 cells were treated with vehicle (DMSO) or 0.25 μM of MEDS433 in the presence of 20 μM uridine alone or in combination with different concentrations of DPY before and during SARS-CoV-2 infection (100 PFU/well). Following virus adsorption, cell monolayers were incubated in the presence of compounds. At 48 h p.i., cell supernatants were harvested and titrated for infectious SARS-CoV-2 on Vero E6 cell monolayers. The data shown represent means ± SD (error bars) of three independent experiments performed in triplicate. Statistical significance was calculated by a one-way ANOVA followed by Dunnett’s multiple comparison test. *** (*p* < 0.0001) compared to the calibrator sample (MEDS433 alone).

The pyrimidine salvage pathway may reduce the antiviral efficacy of a *h*DHODH inhibitor by importing nucleosides from extracellular environment, thus bypassing the block of the de novo biosynthesis [10]. To deal with this problem, the effects of a combination of MEDS433 with an inhibitor of the nucleoside transport, such as dipyridamole (DPY) [32] was investigated. Checkerboard analysis of a MEDS433-DPY combination was therefore carried in Calu-3 cells infected with SARS-CoV-2 in the presence of 20 μM uridine to go beyond its physiological plasma concentrations [33]. As shown in Figure 6, the addition of exogenous uridine reversed the inhibitory effect of 0.25 μM MEDS433 that reduced SARS-CoV-2 replication by more than 80% when tested as single agent (Figure 3C). In contrast, when MEDS433 was examined in the presence of increasing amounts of DPY, the combination of the two molecules restored the antiviral activity of MEDS433 notwithstanding the presence of exogenous uridine (Figure 6). Moreover, the effectiveness of the MEDS433-DPY combination was not due to a reduced Calu-3 cell viability, since none of the tested combinations exerted a cytotoxic effect (data not shown). Together, these results, support the manageability of a combination of a *h*DHODH inhibitor and a pyrimidine salvage inhibitor to inhibit hCoVs replication even in the presence of uridine, thus mimicking in vivo host conditions.

## 4. Discussion

In the last few years, the results of several high-throughput screens to discover broad-spectrum antivirals have identified small molecules targeting the host pyrimidine biosynthesis pathway, mostly with the DHODH activity as their major and specific target [34,35]. These findings highlight the potential of targeting DHODH to design and develop antiviral agents endowed with a high genetic barrier to the development of drug resistance, and the ability to target a broad spectrum of viruses, thus enabling a therapeutic approach of newly emerging viruses and contributing to preparedness for unforeseen viral threats [36].

Given these premises, we have investigated the anti-CoVs potential of a series of new small molecules already selected as potent inhibitors of *h*DHODH activity. Among the tested *h*DHODH inhibitors, the one with the greatest potency, MEDS433 (IC_50_ 1.2 nM), was also observed to be the most effective in inhibiting hCoV-OC43 replication (Figure 1). MEDS433 targets the ubiquinone binding site of *h*DHODH with high affinity, thus reducing the possibility of off-target within the cell [16,17]. This high specificity may therefore explain the extremely high SI values of MEDS433 measured against three different hCoVs in four different cells lines, including normal human lung MRC5 fibroblasts (Table 1). The low level of cytotoxicity is undoubtedly a favorable feature of MEDS433 and indeed contributes to making it a promising candidate to develop a new anti-CoVs antiviral. Under normal physiological conditions, the low proliferating airways epithelial cells satisfy their requirement of pyrimidines mainly through the salvage pathway, which recycles pre-existing nucleosides. In contrast, in hCoVs-infected cells, the exceptional need for large pyrimidines pools to cope with rapid viral replication cannot be supplied sufficiently by nucleoside recycling. It is therefore reasonable that de novo pyrimidine biosynthesis rather than the salvage pathway is more critical for hCoVs replication, effectively making the rate-limiting DHODH enzyme activity crucial for maintaining high levels of viral RNA synthesis. Therefore, the observation that hCoVs replication was prevented by MEDS433 is not surprising. However, it is worth noting that our study adds some new pieces of knowledge that may be of interest to be considered towards the validation of *h*DHODH inhibition as a suitable strategy for the therapeutic management of CoVs infections.

The first consists in the observation of the high potency of MEDS433 against SARS-CoV-2. In this regard, given its critical role for viral replication, *h*DHODH has been considered an emerging target of choice for the development of HTA against SARS-CoV-2 [8,9,14,37]. Thus far, three different *h*DHODH inhibitors have already entered in Phase II/III clinical trials for COVID-19: brequinar (NCT04425252), PTC299 [38] (NCT04439071), and IMU-838 [39] (NCT04379271). The first data available for IMU-838 indicate a clinical activity versus placebo on multiple clinical endpoints (clinical improvement and recovery, reduction of SARS-CoV-2 load in the nasopharynx, systemic anti-inflammatory effects) in hospitalized patients with moderate COVID-19, thus sustaining its further clinical development, as well as the suitability of targeting *h*DHODH for COVID-19 therapeutic intervention [40]. Interestingly, a comparison of the in vitro anti-SARS-CoV-2 efficacy of MEDS433 with that of IMU-838 [31] highlights that MEDS433 is 100-fold more potent than IMU-838 (EC_50_ 7.6 μM, SI 8) with a much more favorable SI (Table 1), thus suggesting for MEDS433 a likely wide therapeutic window of clinical safety and efficacy.

Moreover, the antiviral effect of MEDS433 against SARS-CoV-2 was also observed in the experimental model of kidney organoids (Figure 3D). These organoids are composed of different cell types and model the physiological conditions of the human kidney; thus, they can be used to investigate the effect of SARS-CoV-2 infection on kidney tissues [25,26]. In this regard, we have previously observed that kidney organoids produce infectious SARS-CoV-2 progeny [18]. Although a variability of the effect of MEDS433 on SARS-CoV-2 replication in kidney organoids was measured (Figure 3D), likely reflecting the complexity and heterogeneity of this experimental model [25], the observation of an anti-SARS-CoV-2 activity of MEDS433 even in human organoids is valuable to sustain its preclinical development as a candidate agent for COVID-19.

The second finding that deserves a comment is the antiviral efficacy of MEDS433 against three different hCoVs species. This fact represents an important added value of this new *h*DHODH inhibitor, since in addition to the advantage of overcoming viral drug resistance, it makes MEDS433 of interest for the development of broad-spectrum antiviral agents ready for future emerging CoVs given the independence of its antiviral effects with respect to a specific CoV. We still do not know whether the next emerging CoV may be highly pathogenic as SARS-CoV-1 and MERS, or highly infectious and rapidly spreading as SARS-CoV-2; however, we do know that the possibility exists that novel CoVs will spill-over into human populations, with potentially disastrous consequences [7,36]. Therefore, the need for new broad-spectrum anti-CoVs drugs, effective against both SARS-CoV-2 and CoVs from future zoonoses, is indisputable.

To become a first-line treatment of acute CoV infections, an optimal broad-spectrum CoVs antiviral should, in addition to being highly safe, potent and orally bioavailable, should be suitable for combination treatments that could enhance antiviral efficacy and prevent the emergence of drug resistance [7]. It was therefore pleasing to observe increased efficacy of a combined treatment of MEDS433 with DPY (Figure 6). DPY is a pyrimido-pyrimidine derivative widely used as an oral agent in the prophylaxis of thromboembolism in cardiovascular disease, because of its platelet antiaggregant and vasodilator activities due to the inhibition of the uptake of adenosine into platelets, endothelial cells and erythrocytes [32,41]. DPY is in fact an inhibitor of the equilibrative nucleoside transporters (ENT) 1 and 2, the most effective cell nucleoside/nucleotide transporter involved in the pyrimidine salvage pathway [42]. Indeed, we observed that DPY successfully restored the antiviral activity of MEDS433 concentrations no longer effective against SARS-CoV-2 as a consequence of uridine supplementation mimicking the physiological conditions in the infected host (Figure 6). This finding is relevant for the development of MEDS433 as an antiviral drug candidate, since the failure of some *h*DHODH inhibitors observed in animal models of RNA virus infections is likely due to a systemic compensation of DHODH inhibition by the pyrimidine salvage pathway that flows extracellular uridine into virus-infected cells [43,44,45]. While the combination of DPY with inhibitors of de novo pyrimidine biosynthesis has been investigated previously to increase the anticancer effects of the latter compounds [46], the potential of this combination against virus infections has not yet been thoroughly explored. To this regard, we have recently observed the efficacy of a synergistic combination of MEDS433 with DPY in suppressing the in vitro replication of Herpes simplex virus type 1, even in the presence of hyperphysiological concentration of uridine [47]. Importantly, the DPY concentrations observed to be effective in combination with MEDS433 were lower than the DPY C_max_ [48], and therefore clinically achievable in patients treated with DPY. The wide clinical experience of DPY could allow its rapid repositioning against hCoVs to be clinically useful in combination with HTAs and/or DAAs.

Finally, since *h*DHODH inhibitors have been reported to impact the expression of cytokines and chemokines [14,37] and given the importance of the inflammation state in COVID-19 pathogenesis [1,2], it is tempting to speculate that MEDS433 may be also beneficial on the SARS-CoV-2-associated pathogenic inflammation by affecting the production of proinflammatory cytokines and chemokines. It is therefore possible to hypothesize that MEDS433 may exert a dual-action as candidate therapeutics for COVID-19, not only by inhibiting directly SARS-CoV-2 replication through its ability to cause pyrimidine depletion, but also by alleviating the excessive production and release of proinflammatory cytokines and chemokines. In this scenario, *h*DHODH inhibitors, such as MEDS433, could thus give benefit also in the late stages of COVID-19.

## 5. Conclusions

In conclusion, our study confirms the cellular *h*DHODH activity as a promising target to inhibit SARS-CoV-2 infection [38,39,49], and importantly identifies MEDS433 as an attractive candidate to develop a broad-spectrum anti-CoV HTA that could be rapidly deployable against future novel pathogenic CoVs. For now, the MEDS433′s potent in vitro anti-SARS-CoV-2 activity, and its valuable *drug-like* profile, support further studies to validate its therapeutic efficacy in preclinical animal models of COVID-19.

## Figures and Tables

**Table 1:** Antiviral activity of MEDS433 against different human coronaviruses.

hCoV	Cell Line	EC_50_ (μM) ^a^	EC_90_ (μM) ^b^	CC_50_ (μM) ^c^	SI ^d^
hCoV-OC43	HCT-8	0.012 ± 0.003	0.044 ± 0.021	78.48 ± 4.60	6329
hCoV-229E	MRC5	0.022 ± 0.003	0.288 ± 0.040	104.80 ± 19.75	4763
SARS-CoV-2	Vero E6	0.063 ± 0.004	0.136 ± 0.007	>500	>7900
SARS-CoV-2	Calu-3	0.076 ± 0.005	0.513 ± 0.016	>125	>1600

^a^ EC_50_, compound concentration that inhibits 50% of replication, as determined by FFRA against hCoV-OC43 in HCT-8 cells, or by measuring MRC5 cell viability as a surrogate of viral CPE against hCoV-229E, or by VRAs against SARS-CoV-2 in Vero E6 or in Calu-3 cells. Reported values represent the means ± SD of data derived from three experiments in triplicate. ^b^ EC_90_, compound concentration that inhibits 90% of viral replication. ^c^ CC_50_, compound concentration that produces 50% of cytotoxicity, as determined by cell viability assays in HCT-8, MRC5, Vero E6 or Calu-3 cells. Reported values represent the means ± SD of data derived from three experiments in triplicate. ^d^ SI, selectivity index (determined as the ratio between CC_50_ and EC_50_).

## Data Availability

The data presented in this study are available on request from the corresponding author.
